# Is bone marrow biopsy and aspiration still mandatory when 18F-FDG PET/CT is available for the initial assessment of bone marrow metastasis in pediatric Ewing sarcoma?

**DOI:** 10.3389/fonc.2024.1372481

**Published:** 2024-05-10

**Authors:** Yifei Du, Zhenzhen Zhao, Chao Yang

**Affiliations:** ^1^ Department of Surgical oncology, National Clinical Research Center for Child Health and Disorders, Ministry of Education Key Laboratory of Child Development and Disorders, China International Science and Technology Cooperation Base of Child Development and Critical Disorders, Children’s Hospital of Chongqing Medical University, Chongqing, China; ^2^ Department of Pediatric Surgery, Yibin Hospital Afiliated to Children's Hospital of Chongqing Medical University, Yibin, China

**Keywords:** Ewing sarcoma, bone marrow, PET/CT, pediatric, overall survival

## Abstract

**Purpose:**

To compare the diagnostic value of 18F-fluorodeoxyglucose-positron emission tomography/computed tomography (18F-FDG PET/CT) and bone marrow biopsy and aspiration (BMBA) for evaluating bone marrow metastases (BMM) in newly diagnosed pediatric Ewing sarcoma (ES).

**Material and methods:**

To assess the diagnostic accuracy of 18F-FDG PET/CT against BMBA for marrow infiltration in ES patients, a retrospective analysis encompassed 103 ES patients from the Children’s Hospital of Chongqing Medical University, spanning nine years, who underwent both 18F-FDG PET/CT and BMBA at the point of diagnosis.

**Results:**

The median age of this study was 9.3(15 days to 17.1 years), 52(50.5%) patients were male. Among the cohort, 8 subjects received a BMM diagnosis via marrow cytology or histopathology, concomitant with positive 18F-FDG PET/CT findings. An additional 4 patients were identified with BMM solely through 18F-FDG PET/CT. No cytologically or histologically positive BMM were found in PET/CT-negative patients. Therefore, within this selected sample group, the 18F-FDG PET/CT imaging technique exhibited sensitivity of 100% and specificity of 95.8%. The five-year overall survival rate decreased from 57.5% among the entire cohort of patients to a mere 30% for individuals suffering from BMM.

**Conclusion:**

Given these findings, the prevailing reliance on BMBA warrants reevaluation when 18F-FDG PET/CT is available, potentially heralding a shift towards less invasive diagnostic modalities in the management of ES.

## Introduction

Ewing sarcoma (ES) is a widespread form of cancer and is the second most frequent type of bone tumour among children. It constitutes around 2% of all cancer cases in children and teenagers. Adolescents and young adults are at highest risk for developing ES, which is an aggressive tumor originating from both soft tissues and bone (1). This malignancy often manifests with aggressive behavior and unfavorable survival outcomes, with nearly 30% of newly diagnosed ES patients presenting with distant metastases (2). The most frequent sites of metastasis are the lungs and bone marrow (3). The incidence of bone marrow metastases (BMM) in pediatric ES patients at the time of diagnosis has been reported to range from 5% to 17% (1, 4). Numerous prognostic factors have been identified, including the size of the primary tumor, metastatic status at presentation, and the tumor’s response to chemotherapy (5, 6). Among these factors, metastatic status holds the utmost significance as a prognostic indicator. Patients without metastasis can anticipate a 5-year overall survival (OS) rate of 70-80%. However, in patients with metastatic disease, the OS drops to less than 30%. For patients specifically with bone metastasis and BMM, the OS is less than 10% (1, 7). Moreover, several studies have suggested that BMM itself serves as an independent unfavorable prognostic factor (8, 9). The current risk stratification system, widely used for accurate staging and tailored treatment approaches, emphasizes the critical role of metastatic status.

In the diagnostic workup of ES, several guidelines advocate for a comprehensive suite of evaluations, which typically includes chest computed tomography, magnetic resonance imaging, whole-body bone scans, 18F-fluorodeoxyglucose positron emission tomography/CT (18F-FDG PET/CT), and bone marrow biopsy (BMB). However, these recommendations stem from expert opinion and are with their limitations (10, 11), and optimal combination of imaging modalities was not fully determined (12). At present, BMB is upheld as the definitive standard for the detection and assessment of BMM (13). Despite its status, BMB is an invasive technique and carries an inherent risk of complications, which, although infrequent, include hemorrhage and infection (14). In addition, the accuracy of BMB in detecting ES metastases may be affected by sampling variability, potentially making it less dependable than imaging methods (15).

Currently, 18F-FDG PET/CT is extensively utilized for staging, restaging, and gauging treatment responses in both adult and pediatric oncology. The role of 18F-FDG PET/CT in the assessment of Hodgkin lymphoma is increasingly supported by a growing body of evidence, indicating its potential to replace BMB in this context. However, the efficacy of 18F-FDG PET/CT in detecting BMM and its potential to replace BMB in the initial evaluation of pediatric ES remains an area of ongoing research. The results are varied across different studies, and consensus has yet to be reached regarding the ability of 18F-FDG PET/CT to supplant the bone marrow examination in the initial assessment of ES. The ESMO-PaedCan-EURACAN Clinical Practice Guidelines for bone sarcomas suggest that bone marrow aspiration (BMA) may be omitted if the 18F-FDG PET/CT scan does not indicate metastatic disease (12). Conversely, the National Comprehensive Cancer Network (NCCN) Clinical Practice Guidelines continue to endorse the concurrent use of BMA and 18F-FDG PET/CT scan as part of the standard diagnostic protocol following an ES diagnosis. Meanwhile, the imaging guidelines for children with ES, as proposed by the Children’s Oncology Group Bone Tumor Committee, recommend the 18F-FDG PET/CT scan but do not provide specific directives regarding BMA (11).

The aim of this retrospective study was to evaluate and compare the diagnostic value of 18F-FDG PET/CT to bone marrow biopsy and aspiration (BMBA) of the anterior superior spine(ASIS) in bone marrow infiltration in newly diagnosed ES.

## Materials and methods

After approved by the Review committee of the Children’s Hospital of Chongqing Medical University(CHCMU), we conducted a retrospective analysis of ES cases newly diagnosed from January 2012 to January 2021. CHCMU serves as the premier pediatric medical center for the southwestern region of China, offering healthcare to a population of 200 million in the area, with annual outpatient visits exceeding 3.5 million and inpatient admissions over 100,000. The inclusion criteria as follow: newly histologically confirmed ES patients, and both 18F-FDG PET/CT and BMBA were performed within two weeks. Suspected cases of ES underwent hematological and imaging examinations along with BMBA within two days of being admitted. BMBA, either unilateral or bilateral and blind, was carried out exclusively at the ASIS by a pediatric oncologist, without samples being taken from alternative sites. In cases where the primary tumor originated from the ilium, the side opposite to the primary tumor was preferred for BMBA to investigate bone marrow involvement. The biopsy specimens were assessed by pathologist who was blinded to the PET/CT results. Immunohistochemistry for NKX2.2, as well as CD99, fli1 were conducted to detect morphologically occult tumor cells. In this study, the definitive criteria for BMM diagnosis were marrow cytology and histology. 18F-FDG PET/CT scans were conducted within a week subsequent to the substantiation of the diagnosis. The exclusion criteria were defined as follows: individuals who had previously undergone systemic therapy and those with a time interval exceeding two weeks between the administration of PET/CT and BMBA, as well as cases where either BMBA or 18F-FDG PET/CT was not performed.

18F-FDG PET/CT scans were performed according to a standard whole-body oncological protocol following the guidelines of the European Association of Nuclear Medicine in the affiliated hospital of medical university (16). Radiolabeled FDG was injected intravenously 1 h prior to imaging, and whole body imaging (from the skull to toes) was performed in every patient. The PET/CT images underwent evaluation by two nuclear medicine experts, who were blinded to the results of BMBA. The 18F-FDG PET/CT findings were deemed affirmative when the bone marrow exhibited FDG avidity equivalent to or surpassing that observed in the primary tumor, and also exceeding the avidity detected in surrounding tissues.

18F-FDG PET/CT scans that did not exhibit FDG avidity at any skeletal site were interpreted as negative. Truly negative was defined as both negative of 18F-FDG PET/CT scan and BMBA. The diagnostic precision of BMBA and 18F-FDG PET/CT was evaluated by determining the sensitivity, specificity, as well as the positive predictive value (PPV) and negative predictive value (NPV). The formula to determine these parameters were as following: sensitivity=(True Positives)/(True Positives + False Negatives), specificity=(True Negatives)/(True Negatives + False Positives), PPV=(True Positives)/(True Positives + False Positives), NPV=(True Negatives)/(True Negatives + False Negatives). The data were processed using SPSS software version 26.0 (SPSS Inc., Chicago, IL) and GraphPad Prism version 9.0 (GraphPad Software Inc., La Jolla, CA).Differences between groups were compared using the chi-square tests and Fisher’s exact test for the categorical variables. To assess the concordance between the findings of BMBA and PET/CT, the kappa statistic was employed. Survival analyses were performed using the Kaplan-Meier method with Log-rank test. The level of statistical significance was established for p-values < 0.05.

## Results

**Figure 1:**
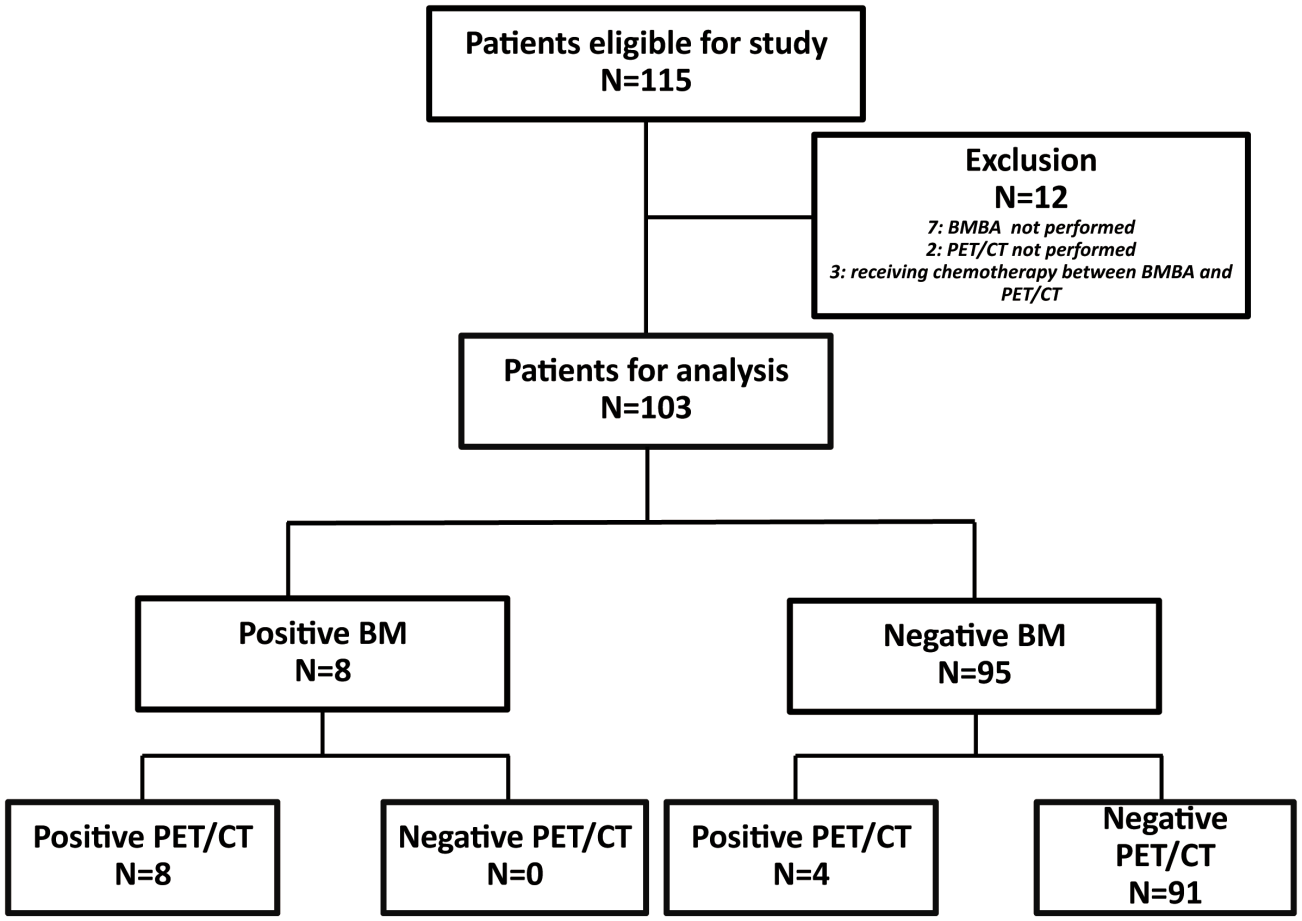
Selection flowchart for this study.

Over the course of nine years, 115 pediatric patients received an ES diagnosis at our institution. As part of the study’s refinement process, seven patients were excluded due to the absence of BMBA. Additionally, two patients were omitted from consideration for lacking 18F-FDG PET/CT scans. Furthermore, an additional three patients were disregarded as they had initiated chemotherapy during the interval between the two aforementioned procedures. Consequently, 103 patients were deemed eligible for inclusion in the study (**Figure 1**).

The demographic and clinical characteristics of our study cohort are showed in **Table 1**. The median age at the time of diagnosis was established at 9.3 years, with a spectrum ranging from 15 days to 17.1 years. 52(50.5%) patients were male. The extremities emerged as the predominant initial site for ES, constituting 33% of the cases within this cohort. Pursuant to the staging criteria delineated by the American Joint Committee on Cancer (AJCC), 50 patients(48.5%) were diagnosed with metastatic disease. The lungs were identified as the most frequent site of metastatic spread, implicated in over half of the metastatic instances in our cohort, affecting 27 patients. A significant majority of the cohort presented with multiple metastatic sites(29/50, 58%).

**Table 1:** Clinical characteristics of the population.

Characteristic	Total (%) n=103
Age at diagnosis (years)	
1-3	13(12.6%)
3-6	18(17.5%)
6-12	44(42.7%)
>12	28(27.2%)
Gender	
Male	52(50.5%)
Female	51(49.5%)
Primary tumor site	
Extremities	34(33.0%)
Trunk	23(22.3%)
Thoracic and abdominal cavity	19(18.4%)
Spine	9(8.7%)
Others	18(17.6%)
Metastatic status	
No metastasis	53(51.5%)
Metastasis	50(48.5%)
Metastatic site	
Bone	17(16.5%)
Lung	27(26.2%)
Lymph node	16(15.5%)
Bone marrow	8(7.8%)
Number of metastatic sites	
1	21(20.4%)
2	14(13.6%)
>2	15(14.6%)
Stage	
I	2(1.9%)
II	12(11.7%)
III	38(36.9%)
IV	51(49.5%)

In discerning bone marrow involvement, BMBA was performed on 70 patients(65.4%), while 33 patients(34.6%) were evaluated with BMA exclusively. The incidence of BMM was corroborated in 8 patients(7.8%) through cytological and/or histological findings. All 8 BMM patients underwent bilateral BMBA, yielding a total of 16 bone marrow results. The details of all the bone marrow results were showed in **Table 2**. It was noted that none of the patients with negative BMB exhibited positive BMA results. BMA and BMB results were in agreement in 13/16 cases(81.3%) (**Table 3**).

**Table 2:** Details about all positive bone marrow results.

Patients	BMB		BMA	
	Left	Right	Left	Right
Positive 1	+	+	+	+
Positive 2	+	+	+	+
Positive 3	–	–	+	+
Positive 4	+	+	+	+
Positive 5	+	+	+	+
Positive 6	–	+	+	+
Positive 7	–	+	–	+
Positive 8	+	–	+	–

+, positive; -, negative.

**Table 3:** Cross-tabulation of BMB versus BMA results for BMM.

BMB status	BMA status	
	Positive	Negative
Positive	11	3
Negative	0	2

The 18F-FDG PET/CT demonstrated bone marrow involvement in 12 cases (11.7%) within our study cohort. **Table 4** provides the correlations between 18F-FDG PET/CT and BMBA findings, stratified by disease subtype. Notably, the 18F-FDG PET/CT scans successfully detected marrow involvement in all 8 patients diagnosed with BMM through cytological or histological assessments. Our study noted an absence of false negative 18F-FDG PET/CT findings; thus, the sensitivity and NPV for 18F-FDG PET/CT stood at 100%. The PPV for 18F-FDG PET/CT in our research was determined to be 66.7%.The kappa statistic measuring the concordance between BMBA and PET/CT was 0.779 (P<0.001), affirming substantial agreement between these two diagnostic tools.

**Table 4:** Cross-tabulation of 18F-FDG PET/CT versus BMBA results for BMM.

18F-FDG PET/CT status	BMBA status	
	Positive	Negative
Positive	8	4
Negative	0	91

In our study, five patients were discovered to have bone marrow lesions beyond the ASIS using PET/CT. It is intriguing to note that the four patients who exhibited positive PET/CT scans but yielded negative results in BMBA, the BMM were observed beyond ASIS region. In contrast, among the 8 patients with both PET/CT and BMBA positive, PET/CT identified additional BMM regions in certain patients beyond for the iliac region.

**Figure 2:**
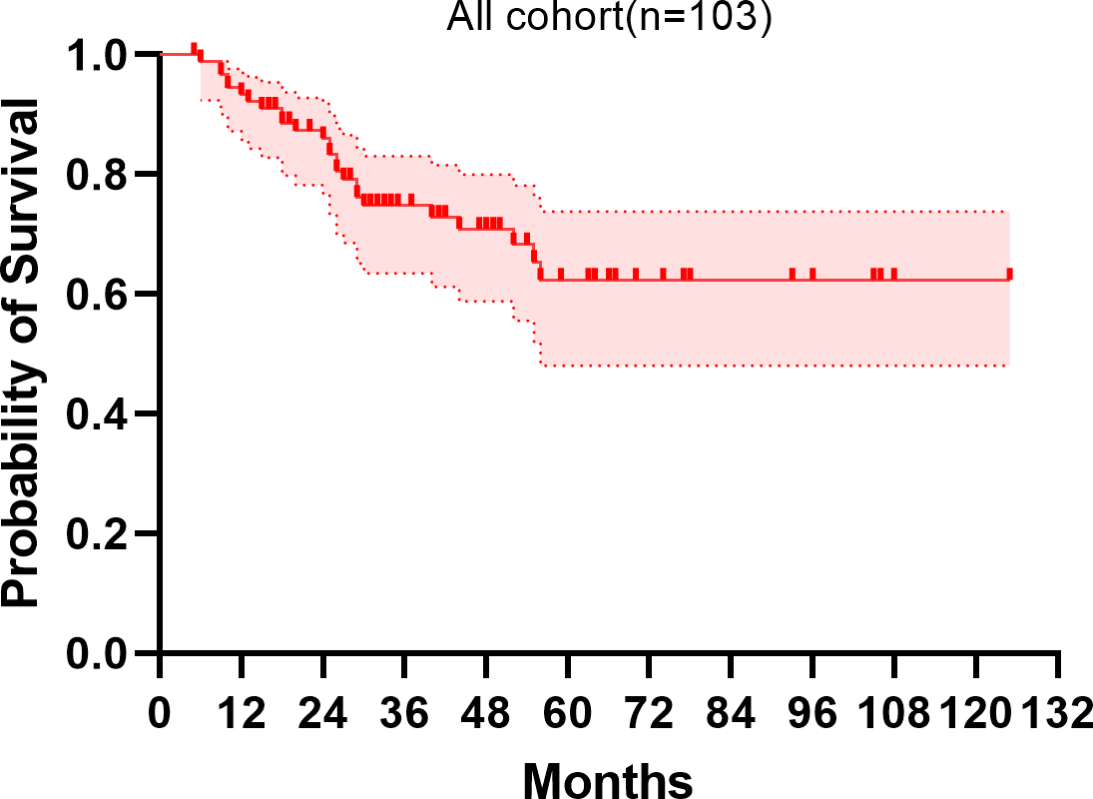
Overall survival of patients.

**Figure 3:**
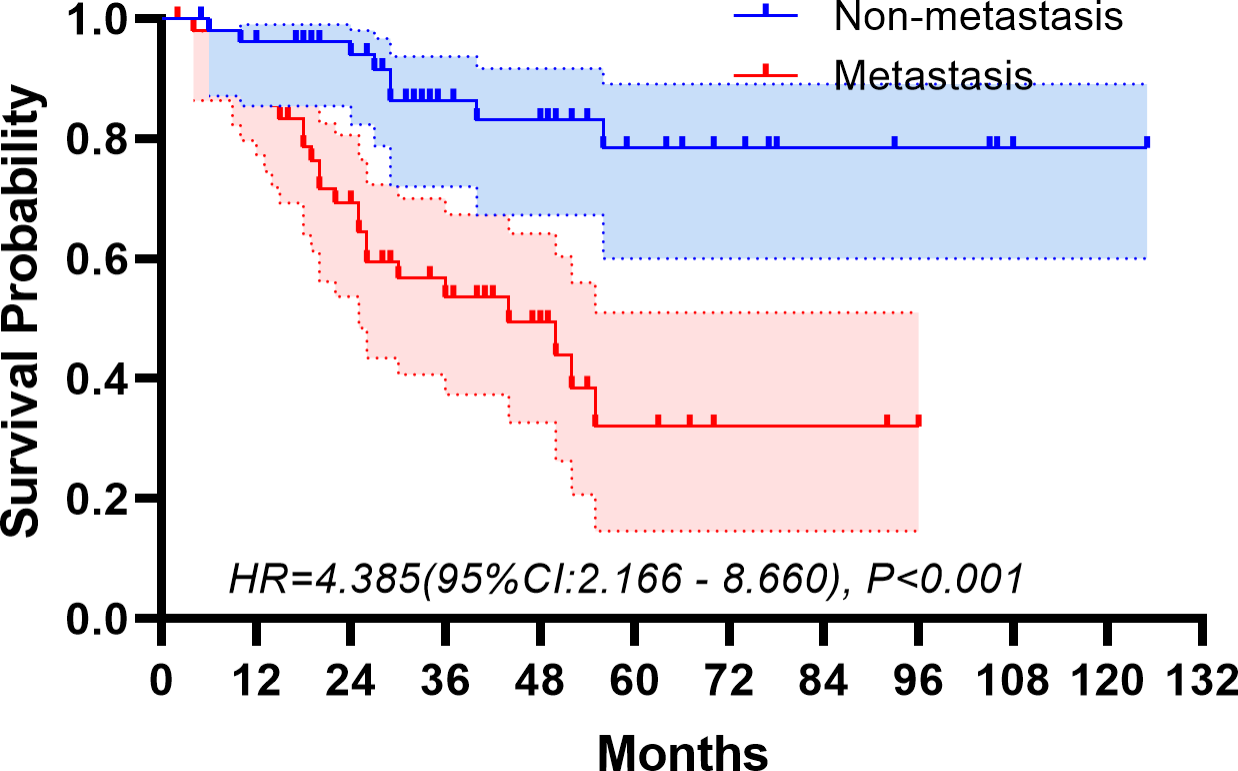
Overall survival of demographic stratified by metastatic status.

**Figure 4:**
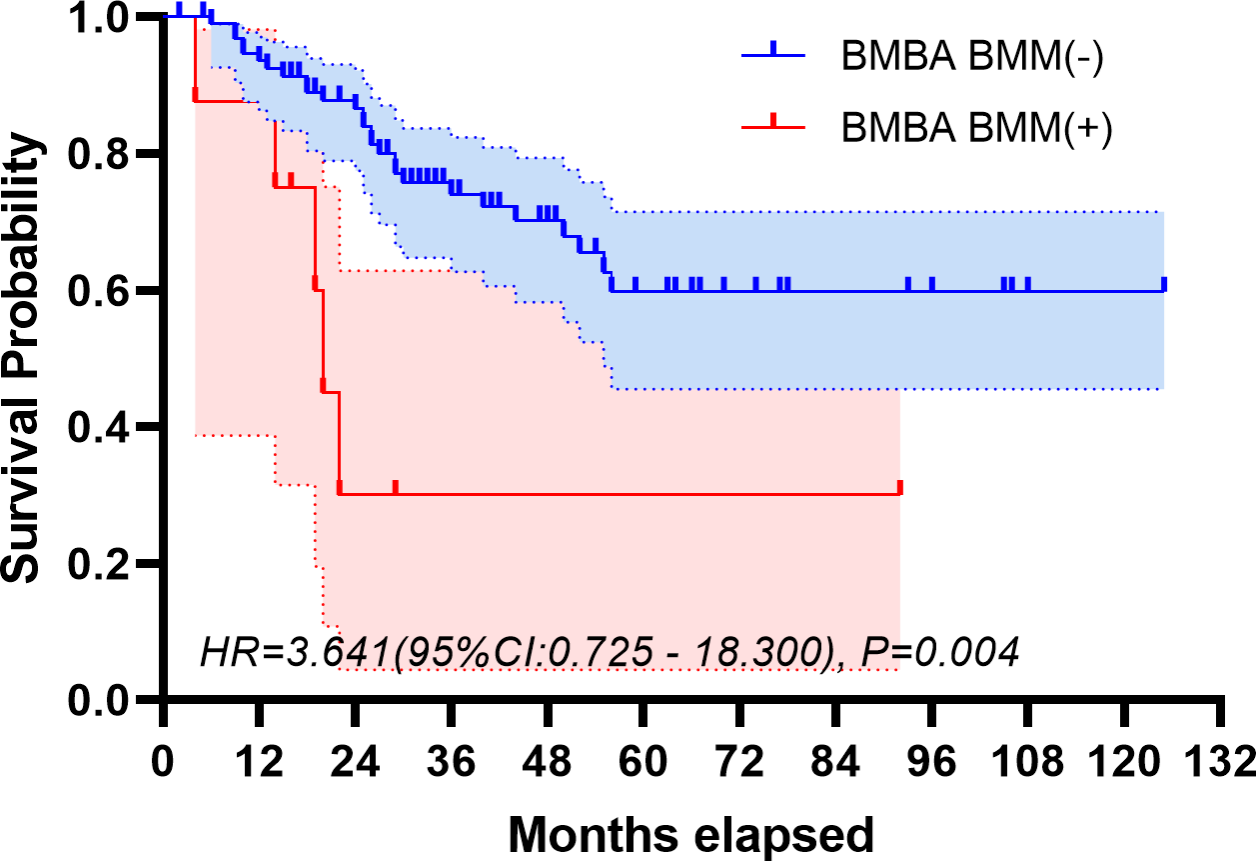
Overall survival of demographic stratified by BMBA-confirmed bone marrow metastatic status.

**Figure 5:**
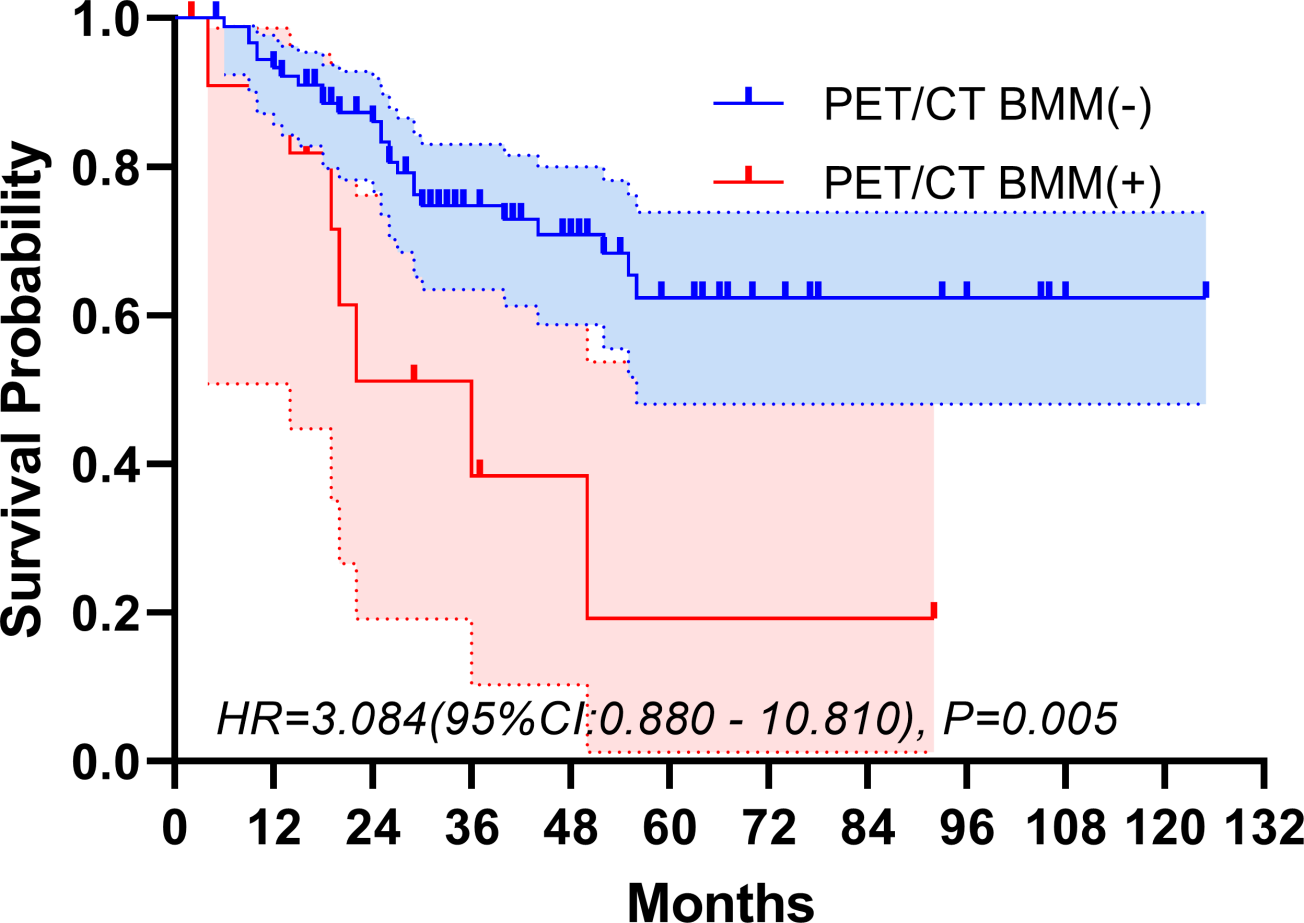
Overall survival of demographic stratified by PET/CT-confirmed bone marrow metastatic status.

The median follow-up period spanned 32 months. The study period witnessed 32 deaths, translating to a mortality rate of 31.1% across the cohort. The five-year overall survival rate was 57.5%. **Figures 2**, **3** depict the survival curves of the entire cohort and the demographic stratified by metastatic status, respectively. Additional survival rate was calculated for patients with BMBA confirmed BMM compared to those identified via PET/CT (**Figures 4**, **5**). Among the BMBA-confirmed BMM patients, 5 individuals died, culminating in a low overall survival rate of a mere 30% within this particular subgroup. In contrast, among 12 patients with PET/CT-positive BMM, the count of mortalities surged to 7, yielding an overall survival rate of merely 19.2% for this cohort.

## Discussion

Bone marrow involvement is a common phenomenon in metastatic ES, often heralded as an independent prognostic marker that portends a less favorable outcome among those afflicted with metastases (8, 9, 17, 18). Thus, its detection is of paramount importance for the initial staging of ES/PNET. BMBA are widely regarded as the diagnostic standard procedures for detecting bone marrow involvement. However, their significance in the initial staging process has yet to be unequivocally elucidated. The likelihood of detecting bone marrow involvement is significantly diminished in the absence of metastases discernable through imaging techniques (19, 20). Contemporary therapeutic protocols stipulate bone marrow examination through biopsy, typically procured from the iliac crest. The underlying assumption of this approach, nonetheless, rests upon the premise that metastasis arising from malignant neoplasms invariably leads to a pervasive infiltration throughout the marrow. Consequently, in instances where BMM is present, a positive biopsy result is anticipated. However, this carries the inherent risk of underestimating cases characterized by a localized bone marrow infiltration pattern (13), potentially leading to an underappreciation of disease severity. Hence, in adult populations, alternative diagnostic means, especially 18F-FDG PET/CT, are frequently utilized to assess BMM. In pediatric population, 18F-FDG PET/CT has been utilized to investigate bone marrow involvement in lymphoma. In spite of this, there is little research evaluating 18F-FDG PET/CT as a means of assessing the bone marrow of pediatric patients suffering from solid tumors.

Contemporary scholarship exhibits a spectrum of views concerning the diagnostic accuracy of 18F-FDG PET/CT in the context of BMM. Tezol et a (21). have raised concerns that 18F-FDG PET/CT may not exhibit the desired levels of sensitivity and precision in pinpointing bone marrow involvement in pediatric neoplasms, inclusive of ES. The above statement is contradicted by findings from other investigations, which propose that 18F-FDG PET/CT’s heightened sensitivity and specificity establish it as a robust tool for evaluating BMM, potentially rendering BMB redundant in the staging of ES. A retrospective analysis conducted by Newman revealed that the incidence rate of BMM detected by BMB was consistent with the results obtained through 18F-FDG PET/CT and bone scan (19). Additionally, Zapata (22) validated the absence of false-negative BMM findings when utilizing 18F-FDG PET/CT in the assessment of pediatric solid tumors. Supporting these findings, Kasalak (20) reported a substantial concordance rate of 95% between 18F-FDG PET/CT and BMB in diagnosing BMM.

In this retrospective investigation, we assessed and compared the diagnostic efficacy of 18F-FDG PET/CT with BMBA in BMM among newly diagnosed ES children and adolescent. To the best of our knowledge, this study is the largest cohort assessing the efficacy of 18F-FDG PET/CT in BMM in pediatric ES, involving a total of 103 participants. A high sensitivity (100%) and specificity (95.8%) of 18F-FDG PET/CT for the detection of marrow disease in pediatric ES were observed, which is consistent with the results obtained in previous studies (20, 23). A systematic review conducted by Campbell (24) to assess the role of BMB for staging in ES inferred that BMB might no longer be necessary for the staging of ES. In a substantial retrospective cohort study comprised of 180 patients with ES, conducted by the French Sarcoma Group (25), the precision of 18F-FDG PET/CT in the detection of BMM at the point of diagnosis was critically evaluated. The findings revealed a sensitivity of 92.3% and an impressive specificity of 99.4%, thereby attesting to the superior efficacy of 18F-FDG PET/CT over BMBA for the appraisal of bone marrow infiltration. Complementary to this, an additional study has shed light on the relatively minimal diagnostic benefit of routine BMBA usage in the staging process of extraskeletal ES. It was elucidated that BMBA was not consistently effective in diagnosing metastatic engagement, even in cases where bone metastases were already known (26).

In this study, we also analyzed 8 patients diagnosed with BMM through histology and cytology. Each of the 8 patients underwent bilateral BMBA, resulting in a total of 16 BMA and BMB samples. Among these 16 samples, the concordance between BMA and BMB was 81.3% (including 11 concordant positive results and 2 concordant negative results). On a patient level, regardless of which side (left or right) was positive, it was considered as BMM, resulting in a concordance of 87.5%(7/8). In a study compared the results of BMA and BMB, 61.25% of the cases showed a positive correlation between BMA and BMB (27). In another reports, there was only 22% positive correlation in the findings on aspirates and biopsies (28). Our results were significantly higher. However, inconsistencies still existed between the left and right side results as well as between BMA and BMB results. Therefore, bilateral bone marrow examination remains the standard procedure for assessing bone marrow status in patients with proven or suspected malignancies.

Beyond the matter of discerning positivity rates for BMM, there are other considerations that sway the selection of diagnostic modalities by healthcare providers and their patients. It is widely acknowledged that 18F-FDG PET/CT is a financially demanding procedure, incurring a cost upwards of $800 in China, whereas BMBA imposes a considerably milder economic impact, priced below $150 – a factor which significantly lightens the financial load on patients. Contrastingly, in the United States, the fiscal implications of BMBA and 18F-FDG PET/CT are more pronounced, with associated expenses ranging from $500 to $1,500 for BMBA (22), and about $1600 for 18F-FDG PET/CT (29, 30), placing healthcare costs at the forefront of decision-making for both physicians and patients when deliberating the use of PET scans versus BMBA. Moreover, the invasive nature of BMBA bears the risk of bleeding and often necessitates sedation or general anesthesia, particularly in pediatric cohorts, to mitigate the pain and distress it might induce. In contradistinction, 18F-FDG PET/CT is a non-invasive investigative tool which, besides negating the potential for BMM, yields a broader spectrum of clinical data, thereby enhancing its overall clinical utility.

In consideration of the vast expanse of literature available to us and the understandings we have gleaned from our investigations, we propose a re-evaluation of the traditional invasive BMBA as a means of staging ES at the time of diagnosis. Our recommendation is that BMBA should no longer be a mandatory systematic procedure in this particular clinical scenario, instead, we advocate for the adoption of non-invasive 18F-FDG PET/CT, which has evidenced remarkable diagnostic efficacy. Embracing this paradigm shift holds the potential to streamline the staging process and alleviate the burden placed upon patients.

This study is subject to several limitations that warrant acknowledgment. Firstly, it is important to note that this is a retrospective study conducted at a single center, which may limit the generalizability of the findings. Secondly, the evaluation of results from alternative imaging modalities was not included in our analysis, which could potentially provide valuable insights. And we did not perform BMBA under PET/CT guidance, so we cannot verify whether the FDG uptake on PET/CT was due to true BMM. Additionally, it is worth mentioning that bone marrow cytology tests, which we relied on, have inherent limitations and may have a certain rate of missed diagnoses. Furthermore, the detection of tumor DNA in bone marrow using PCR or NGS methods was not employed in our study, and incorporating these advanced techniques could enhance the accuracy of our findings. Finally, it should be acknowledged that we solely focused on the ASIS region for BMBA and did not perform image-guided BMBA at noniliac lesions exhibiting enhanced FDG uptake, which may have implications for the comprehensiveness of our results.

## Conclusion

In conclusion, the utilization of 18F-FDG-PET/CT represents a highly valuable approach in the evaluation of bone marrow involvement in newly diagnosed ES. The conventional practice of indiscriminately conducting BMBA of the iliac crest should be reevaluated in light of the availability and effectiveness of 18F-FDG-PET/CT imaging.

## Data availability statement

The original contributions presented in the study are included in the article/supplementary material. Further inquiries can be directed to the corresponding author.

## Ethics statement

The studies involving humans were approved by The Review committee of the Children’s Hospital of Chongqing Medical University. The studies were conducted in accordance with the local legislation and institutional requirements. Written informed consent for participation in this study was provided by the participants’ legal guardians/next of kin. Written informed consent was obtained from the individual(s), and minor(s)’ legal guardian/next of kin, for the publication of any potentially identifiable images or data included in this article.

## Author contributions

YD: Investigation, Supervision, Writing – original draft, Writing – review & editing. ZZ: Resources, Writing – original draft, Writing – review & editing. CY: Conceptualization, Methodology, Supervision, Writing – original draft, Writing – review & editing.
